# Selenium intakes in the Irish adult population

**DOI:** 10.1017/jns.2023.23

**Published:** 2023-03-13

**Authors:** Maria Buffini, Anne P. Nugent, Janette Walton, Albert Flynn, Breige A. McNulty

**Affiliations:** 1 UCD Institute of Food and Health, School of Agriculture and Food Science, University College Dublin, Belfield, Dublin 4, Republic of Ireland; 2 Institute for Global Food Security, Queens University Belfast, Belfast, Northern Ireland, UK; 3 School of Food and Nutritional Sciences, University College Cork, Cork, Republic of Ireland; 4 Department of Biological Sciences, Munster Technological University, Cork, Republic of Ireland

**Keywords:** 4-d food diary, Adults, Ireland, Nutrition surveys, Selenium intake

## Abstract

Selenium (Se) is an essential trace element which has an important role as a constituent of seleno-proteins involved in various physiological processes. Previous research in Irish adults suggests that intakes of this important nutrient are suboptimal. The aim of the present study was to estimate the current intakes and major food sources of Se by Irish adults. Mean daily intakes (MDIs) of Se were calculated using data from the National Adult Nutrition Survey which involved 1500 Irish adults aged 18–90 years. The Se content of foods and drinks consumed over a 4-d period was determined using data from the Irish Total Diet Study (TDS). Adequacy of Se intakes was assessed by calculating the proportion of the population with intakes below the adequate intake (AI) of 70 μg/d and lower reference nutrient intake of 40 μg/d (LRNI). The MDI of Se in the total population was 71⋅7 μg/d, with significantly higher intakes reported in men (80⋅2 μg/d) compared with women (63⋅4 μg/d, *P* < 0⋅01). Meat and meat products were the major contributing food group to Se intakes for both men (37 %) and women (31 %). Overall, 47 % of the population were not meeting the recommended AI, while 4 % of the total population were not meeting the LRNI. Although the average intake of Se is above the AI, a significant proportion of the population is not meeting this recommendation and continued monitoring of Se intakes is necessary, particularly by at-risk groups and also in the context of sustainability.

## Introduction

The mineral selenium (Se) is distributed widely in nature and is found in most rocks and soils at concentrations between 0⋅1 and 2⋅0 ppm^(1)^. Soluble selenates are readily taken up by plants and converted to organic compounds such as selenomethionine and selenocysteine^(1)^. In humans, this essential trace element is a component in the structure of seleno-proteins which are required for human health and plays a role in several physiological processes. Approximately thirty-five seleno-proteins have been identified, the functions of which are wide ranging and include a role in maintenance of fertility, immunity and thyroid hormone regulation^(2,3)^.

Dietary intake of Se affects the efficacy of the seleno-proteins, and thus, the efficiency of the physiological processes that they are required for. Hence, inadequate Se intake can have adverse health effects, with evidence suggesting that it may be associated with diseases such as cancer, diabetes and cardiovascular disease (CVD)^(4)^. This inverse relationship between selenium status and CVD risk is potentially due to Se having an antioxidant function via the enzyme glutathione peroxidase, as well as other seleno-enzymes^(5)^. Moreover, Se deficiency is thought to have a deleterious effect on immune function; this link is quite poignant at present given the recent COVID-19 pandemic. There is a growing body of research suggesting that deficiencies in important micronutrients such as Se are evident in patients with acute respiratory tract infections such as COVID-19^(6,7)^.

Se is present in relatively high amounts in a variety of sources including fish, offal, brazil nuts, eggs and cereals^(8)^. Nonetheless, low Se consumption has historically been an issue in many European populations, and interestingly, a gradient does seem to exist across Europe with Eastern populations having notably lower intakes, compared with that of Western European populations^(9)^. Intakes as low as 25 μg/d have been reported in a Polish cohort, and intakes in Italy and Slovenia have also been found to be below the recommended dietary allowance (RDA) of 55 μg/d^(5)^, while studies involving Spanish populations often exceeded the RDA, with the highest intakes reported in Spanish men at 107⋅1 μg/d^(9)^. A previous report on Se intakes by the Irish population in 2002 reported that 45⋅6 % of women and 17⋅1 % of men had intakes of Se below the average requirement (AR) of 40 μg/d^(10)^. Correspondingly, previous studies reviewing Se status in Europe have shown that serum Se levels are below the optimal 100 μg/l in seven European countries^(11,12)^. More recent reports of Se status have corroborated this, by suggesting that suboptimal Se status is widespread throughout Europe^(9)^. The amount of Se available in the soil for plant growth and corresponding variations in the intake of Se by humans, differs considerably among regions and countries^(13,14)^. Reductions in the use of Se-rich wheat imported from the USA and Canada, and increase usage of European, which has a poorer Se content, has in the past been suggested as a possible cause of declining Se intakes and status in the UK and other European countries^(15)^. The cost of importing wheat is set to rise due to Brexit and many countries, including Ireland, are striving to achieve a more sustainable diet. Subsequently, manufacturers are sourcing more locally available commodities such as wheat, the European varieties of which have a lower Se content, it is important to monitor baseline intakes of micronutrients like Se which are susceptible to such economic changes.

The primary aim of the present study was to examine Se intakes of the Irish adult population in a recent dataset, and to explore the different patterns of intake between age and gender subgroups. A second objective was to investigate the dietary sources of Se in a bid to inform policy makers of the most important contributing food groups within the Irish diet by comparing the consumption patterns of low Se consumers to those of high Se consumers.

## Methods

### Study population

The National Adult Nutrition Survey (NANS) is a cross-sectional food survey, completed between 2008 and 2010, and conducted by the Irish Universities Nutrition Alliance (IUNA). A more detailed description of the survey methodology including sampling frame, eligibility criteria and response rates has been reported previously^(16)^. A database of names and addresses held by Data Ireland (National Postal Service) was used to randomly select persons in 20 geographical clusters across the country, selected to provide proportional representation of urban–rural divide. In total, 1500 free living adults aged 18–90 years (740 men and 760 women) took part in the survey with a response rate of 60 %. There were few exclusion criteria, other than pregnancy/lactation and inability to complete the survey because of disability. The sample was representative of the Irish population with respect to gender, age group, geographical location and social class as per the 2006 Irish census (www.cso.ie). In addition to food and beverage intake data, anthropometric, socio-demographic, health and lifestyle, and physical activity data were also collected. This non-dietary data was collected by means of questionnaires which the participant themselves completed, with the exception of the anthropometric data which was collected by trained researchers^(16)^. Any demographic variables with missing values have been highlighted in Table 3. This study was conducted according to the guidelines laid down in the Declaration of Helsinki and all procedures involving research study participants were approved by the University College Cork Clinical Research Ethics Committee of the Cork Teaching Hospitals and the Human Research Committee of University College Dublin (ECM 3 (p) 4 September 2008). Written informed consent was obtained from all participants.

### Dietary assessment

Food and beverage intake was determined using a 4-d semi-weighed food diary and assessed using WISP V3.0 (Tinuviel Software, Anglesey, UK), based on data from the McCance and Widdowson's The Composition of Foods 5th and 6th editions plus supplemental volumes, to calculate nutrient intakes^(17–26)^. Each food/beverage consumed was recorded and allocated an individual food code and brand code with the participant recording the time of eating, the eating location and self-defining each eating occasion as either a meal or a snack. Adjustments were made to the food composition database and supplemented with Irish food codes to take account of recipes, nutritional supplements, commonly consumed generic Irish foods and new foods on the market. The database generated from the food and beverage intake data comprised 133 050 rows of data. Each row of data described each food and drink item along with its nutritional content consumed by all NANS participants at every eating occasion throughout the 4 d. A total of 2552 food codes were consumed during the survey; each food code was allocated to one of 19 food groups. The nineteen food groups examined in this study have been outlined previously^(16)^.

### Updating of selenium composition

The Se composition of foods within the Irish food composition database was deemed to be uncertain, as the majority of Se data were derived from the McCance and Widdowson composition of foods. The initial dataset, which is based on food available in the UK, is unlikely to fully represent Se occurrence in Ireland, as Se content of food varies greatly depending on the soil where the food has been grown. In addition, the database had not recently been systemically updated for Se content, and therefore was unlikely to provide a reliable basis for the assessment of Se intake. Therefore, work was carried out to provide accurate estimates of the Se composition of foods consumed within the NANS. Firstly, an SPSS database was created containing all of the 2552 NANS foods consumed, including recipes. Secondly, this database was examined on a food code-by-food code basis, and each food code was assigned an Se concentration (μg/100 g) on the basis of analytical data and other published data sources^(17–28)^. All Se containing foods, drinks and recipes were identified and a new Se database was formed, with only approximately 9 % of all food codes assigned a value of 0 μg/100 g (mostly nutrient-specific supplements e.g. vitamin C). The Se content of foods and drinks was predominantly determined using Se data from the Total Diet Study (TDS) (87 %)^(27)^. For certain food codes where Se values were not available, the UK food composition tables (<1 %)^(17–26)^ and the US food composition tables (<4 %)^(28)^ were consulted to assign suitable values. For composite dishes and recipes, which accounted for approximately 17 % of food codes examined, the Se content of each ingredient was identified and calculated as a proportion of the total weight of the dish to give an overall Se amount for that dish.

### Statistical analysis

Statistical analyses were carried out using IBM® SPSS® V26.0 statistical software package version 24.0 (SPSS Inc., Chicago, IL, USA). The MDI of Se was calculated for the total population and separately for men and women, per age group. Se intakes from all sources i.e. including nutritional supplements, as well as food sources only, were examined. The mean daily intake (MDI) of Se in men *v*. women were analysed using an unpaired *t* test. Differences in intakes between age groups, within each gender were evaluated using a one-way analysis of variance ANOVA with Bonferroni *post-hoc*. Energy under-reporters were identified by evaluating reported energy intake (as a ratio of energy intake to basal metabolic rate (EI:BMR)) against presumed age-specific energy cutoffs calculated on the basis of reported levels of physical activity^(29,30)^. To examine the effect of geographical location on Se consumption, specific categories were created as follows: location was classified into three categories, small towns, large towns and cities (populations of <9999; >10 000 and cities, respectively). According to the European Food Safety Authority (EFSA), there is insufficient data from human studies to derive an AR for selenium, thus Se intakes by NANS participants were examined in terms of the adequate intake (AI) level. The proportion of the population (excluding under-reporters) with intakes below the AI of 70 μg/d and lower reference nutrient intake of 40 μg/d (LRNI) were calculated^(31,32)^. The proportion of the population consuming excess Se in their diets i.e. above the most conservative tolerable upper limit (TUL) set for Se i.e. 300 μg/d^(33–35)^ was also examined. Men and women were separated into tertiles based on their average daily Se intakes, and these tertiles were examined for differences in demographic characteristics. Alternate Healthy Eating Index (AHEI) scores were assigned based on the criteria of McCullough *et al.*, with a higher overall score representing a healthier diet pattern^(36)^. General linear models (GLMs) adjusting for age group (men only), supplement use (women only) and underreporting were carried out in order to compare micronutrient intakes of tertile members. A Bonferroni correction was applied by adjusting the *P*-values by the number of traits in each table. *P*-values that exceed 1⋅0 after correction for multiple testing have been marked down to 1⋅000.

## Results

The MDI of Se by the total population, from all sources (food and supplements) was 71⋅7 ± 30⋅2 μg/d, and from food sources only 68⋅5 ± 25⋅7 μg/d (mean ± sd, Table 1). Se intake was predominantly from food sources only i.e. excluding nutritional supplements, for both genders (97 % for men and 93 % for women). The MDI of Se from all sources was significantly higher in men of all ages (80⋅2 ± 29⋅1 μg/d) when compared with women (63⋅4 ± 28⋅9 μg/d, *P* < 0⋅01). Similarly, Se intakes from food only were higher in men compared with women at 78⋅1 ± 26⋅9 μg/d and 59⋅1 ± 20⋅7 μg/d, *P* < 0⋅01, respectively. Differences in Se intakes were observed across age groups for men, with the youngest men (18–35 years) having significantly higher intakes in comparison to older male age groups for both all sources and food sources only (*P* < 0⋅01, Table 1). This contrasted with women, where no differences were noted between age groups. Interestingly, when adjusted for energy, results showed that Se intakes from all sources were higher in women than men at 90⋅9 ± 40⋅4 μg/10 MJ/d *v*. 83⋅7 ± 24⋅8 μg/10 MJ/d (*P* < 0⋅01, Supplementary Table S1). Similarly, Se from food sources only, were higher in women than men at 84⋅7 ± 26⋅2 μg/10 MJ/d and 81⋅5 ± 22⋅6 μg/10 MJ/d.

**Table 1.** Mean daily Se intake (μg) from all sources (food and supplements) and from food sources only, split by gender and by age group

	Total population (*n* 1500)	Men (*n* 740)	Women (*n* 760)	Men				Women			
				18–35 years (*n* 276)	36–50 years (*n* 205)	51–64 years (*n* 153)	≥65 years (*n* 106)	18–35 years (*n* 255)	36–50 years (*n* 232)	51–64 years (*n* 153)	≥65 years (*n* 120)
All sources (μg)											
Mean	71·68	80·20	63·38*	86·28^a^	76·84^b^	77·39^b^	74·92^b^	63·75	62·29	65·25	62·28
sd	30·19	29·13	28·87	34·12	23·81	24·27	28·52	26·07	24·41	33·11	36·05
Median	66·89	75·56	57·68	81·05	74·44	43·42	71·13	59·31	56·86	59·11	54·28
P5	35·48	42·64	31·92	44·21	47·54	73·00	40·34	29·49	34·12	33·30	31·06
P97·5	148·16	155·24	127·89	167·89	138·77	138·49	156·86	125·97	119·46	139·02	185·04
Food sources (μg)											
Mean	68·47	78·05	59·13*	83·49^a^	75·41^b^	75·33^b^	72·93^b^	61·27	58·61	59·30	55·38
sd	25·74	26·88	20·70	32·20	22·03	19·97	26·85	24·46	18·63	17·97	18·59
Median	65·14	74·96	56·13	79·45	73·82	73·00	70·77	57·83	55·70	58·16	52·89
P5	35·23	42·43	31·75	43·45	47·54	43·42	38·34	29·26	34·12	33·30	31·03
P97·5	126·71	147·45	109·65	167·36	135·92	122·95	156·86	124·78	109·37	100·77	109·47

P, percentiles. Significant differences (*P* < 0·01) between age groupings within the same gender are compared by ANOVA and are denoted by superscript letters, while the * denotes a significant difference between men and women compared by a *t* test.

The percentage of participants whose MDI did not meet the EFSA recommended AI level for Se of 70 μg/d was calculated for valid reporters only^(31)^. From Table 2, it is apparent that overall 47 % of the total population were not meeting this recommendation with 65 % of women and 29 % of men found to have intakes that were below the AI. For both genders, the over 65 s had the highest proportion of those not meeting the recommended AI. In addition, 7 % of women and 1 % of men had mean daily intakes which fell below the LRNI of 40 μg/d^(32)^, again the highest proportion of these being in the over 65 age group. No one was found to have exceeded the TUL for Se of 300 μg/d^(33)^.

**Table 2.** Percentage of population groups with mean daily Se intakes μg/d from all sources below the adequate intake and lower reference nutrient intake and above the tolerable upper limit

	Total population^a^ (*n* 1051)	Men (*n* 523)	Women (*n* 528)	Men				Women			
				18–35 years (*n* 207)	36–50 years (*n* 143)	51–64 years (*n* 98)	≥65 years (*n* 75)	18–35 years (*n* 170)	36–50 years (*n* 165)	51–64 years (*n* 106)	≥65 years (*n* 87)
% ↓AI	46·81	28·68	64·77	20·77	34·27	30·61	37·33	57·06	67·27	66·98	72·41
% ↓LRNI	4·09	0·96	7·20	0·00	2·10	0·00	2·67	7·65	5·45	6·60	10·34
% ↓TUL	0·00	0·00	0·00	0·00	0·00	0·00	0·00	0·00	0·00	0·00	0·00

AI (adequate intake), LRNI (lower reference nutrient intake) and TUL (tolerable upper limit) are 70, 40 and 300 μg/d, respectively^(31–33)^.

a Under-reporters excluded.

**Fig. 1.**
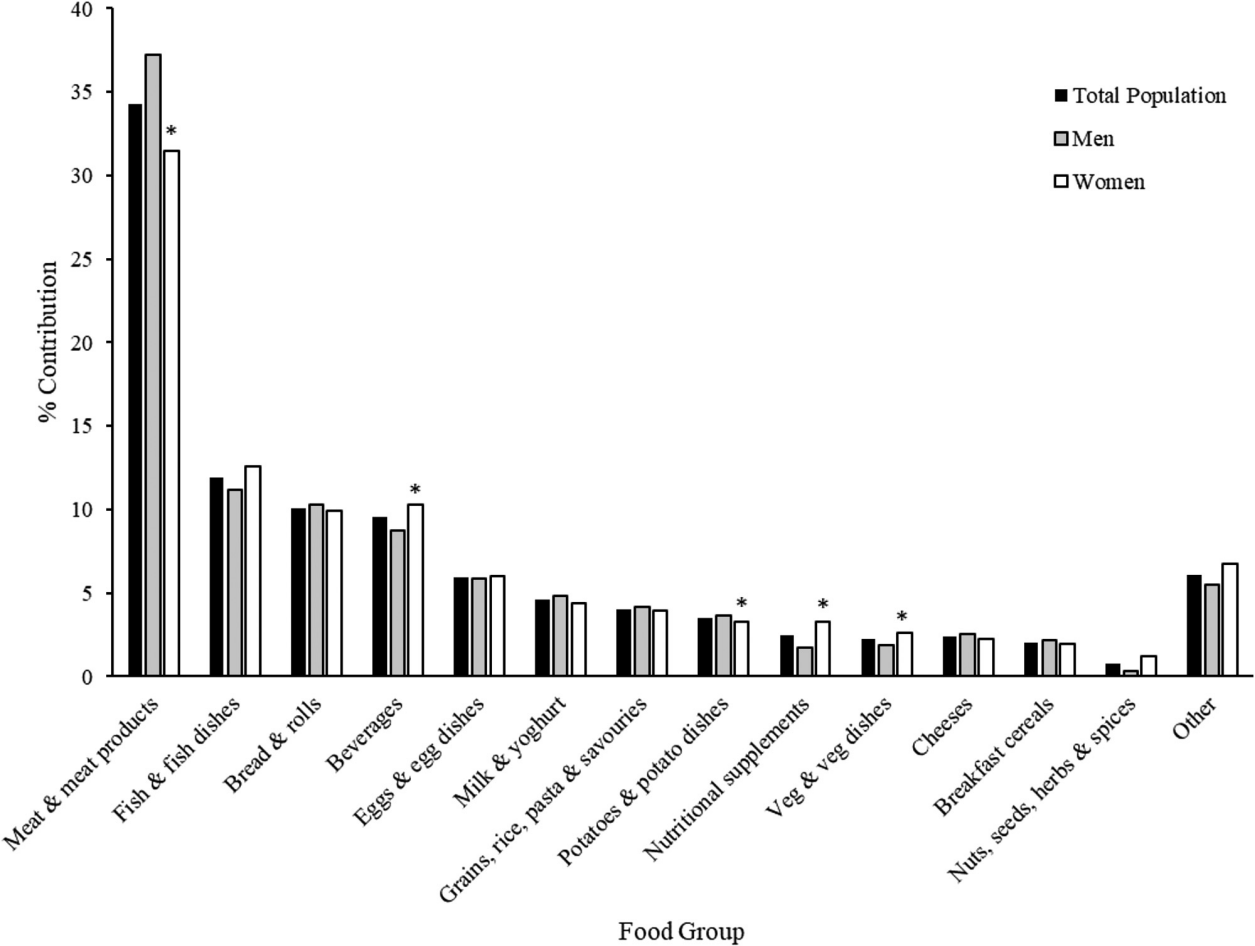
Percentage contribution of main food groups to mean daily Se intakes for men and women. *Denotes significant differences (*P* < 0⋅05) in the contributing food groups between men and women when compared with a *t* test. The ‘other’ category included ‘Biscuits, cakes and pastries’, ‘Soups and sauces’, ‘Fruit and fruit dishes’, Sugars, confectionary and preserves’, ‘Ice-creams and desserts’, ‘Nuts and seeds’ and ‘Spreading fats and oils’.

The main food groups contributing to Se intake are displayed in Fig. 1. Meat and meat products were the main contributor to Se intakes for both men and women at 37 and 31 %, respectively, this was followed by fish and fish dishes at 11 and 13 %, respectively. The third largest contributor for men were bread and rolls at 10 %, while for women it was beverages at 10 %. The IUNA food group ‘beverages’ is a very broad category, and the contributions of each beverage subcategory contributed the following to overall beverage consumption; 35 % ‘other beverages’ (flavoured and unflavoured water, hot water-based drinks made up with cappuccino powder, for example), 30 % ‘teas’, 20 % alcoholic beverages, while the final 15 % includes coffees, carbonated beverages, squashes and cordials. As previously inferred, nutritional supplements contributed more to Se intake for women compared with men (3·3 and 1·7 %, respectively). In order to profile the consumption patterns of low, medium and high Se consumers, the data were spilt into tertiles, separately for each gender. In Table 3, the demographic characteristics across these tertiles are described. There was no significant difference between the social class, location or smoking status of participants across the tertiles of Se intake, for either gender. There were, however, notable differences in age and body mass index (BMI) between low, medium and high male consumers, with the high Se consumer group being the youngest at 41 years (*P* < 0·001) and having the lowest BMI's at 26·9 kg/m^2^ (*P* = 0·028). There was a significant difference in supplement use between low, medium and high female consumers and not surprisingly, the highest proportion of these at 64 % were in the high Se consumer group (*P* < 0·001). Both genders had a significantly higher proportion of under-reporters in the low Se consumer group (*P* < 0·001). Differences in age, supplement use and underreporting were adjusted for during any subsequent tertile analysis.

**Table 3.** Demographic characteristics of tertile groups (low, medium and high Se consumers) split by gender

Demographic	Men				Women			
	Low Se (*n* 246)	Medium Se (*n* 247)	High Se (*n* 247)	*P*-value	Low Se (*n* 253)	Medium Se (*n* 254)	High Se (*n* 253)	*P*-value
Age in years (mean ± sd)	46·60^a^ ± 17·44	44·05^a^ ± 16·62	40·80^b^ ± 17·17	**<0·001**	46·30 ± 17·77	45·11 ± 16·65	44·07 ± 16·08	0·330
Social class* (%)								
Professional	45·92	42·21	45·31	0·112	46·72	48·52	51·43	0·522
Non-manual skilled	19·74	16·80	10·53		20·09	21·52	23·27	
Manual skilled	15·45	18·85	19·83		11·35	13·08	10·20	
Unskilled	18·84	21·13	24·29		21·83	16·88	15·10	
Location^†^ (%)								
Small town <9999	28·86	35·22	31·98	0·237	33·99	32·68	39·92	0·280
Large town	41·46	31·58	38·06		35·97	33·07	28·49	
City (Dublin/Cork)	29·67	33·20	29·96		30·04	34·25	31·62	
BMI* (kg/m^2)^	27·93^a^ ± 4·39	27·89^a^ ± 4·52	26·94^b^ ± 4·55	**0**·**028**	27·23 ± 5·66	26·21 ± 4·62	26·27 ± 5·58	0·064
Smokers* (%)	21·01	21·63	17·89	0·542	25·00	20·72	16·67	0·072
Supplement users* (%)	34·87	39·43	45·34	0·061	41·70	48·40	64·29	**<0**·**001**
Under-reporters* (%)	60·48	25·81	15·67	**<0**·**001**	59·05	29·07	18·10	**<0**·**001**

* Excludes some missing values. *P*-values refer to differences across demographics which were assessed by *χ*^2^ tests (categorical variables) or ANOVA (continuous variables). ^a,b^Mean values with unlike superscript letters are significantly different between age and BMIs of male tertiles (*P* < 0·05 have been highlighted in bold font).

† Refers to the number of inhabitants.

**Fig. 2.**
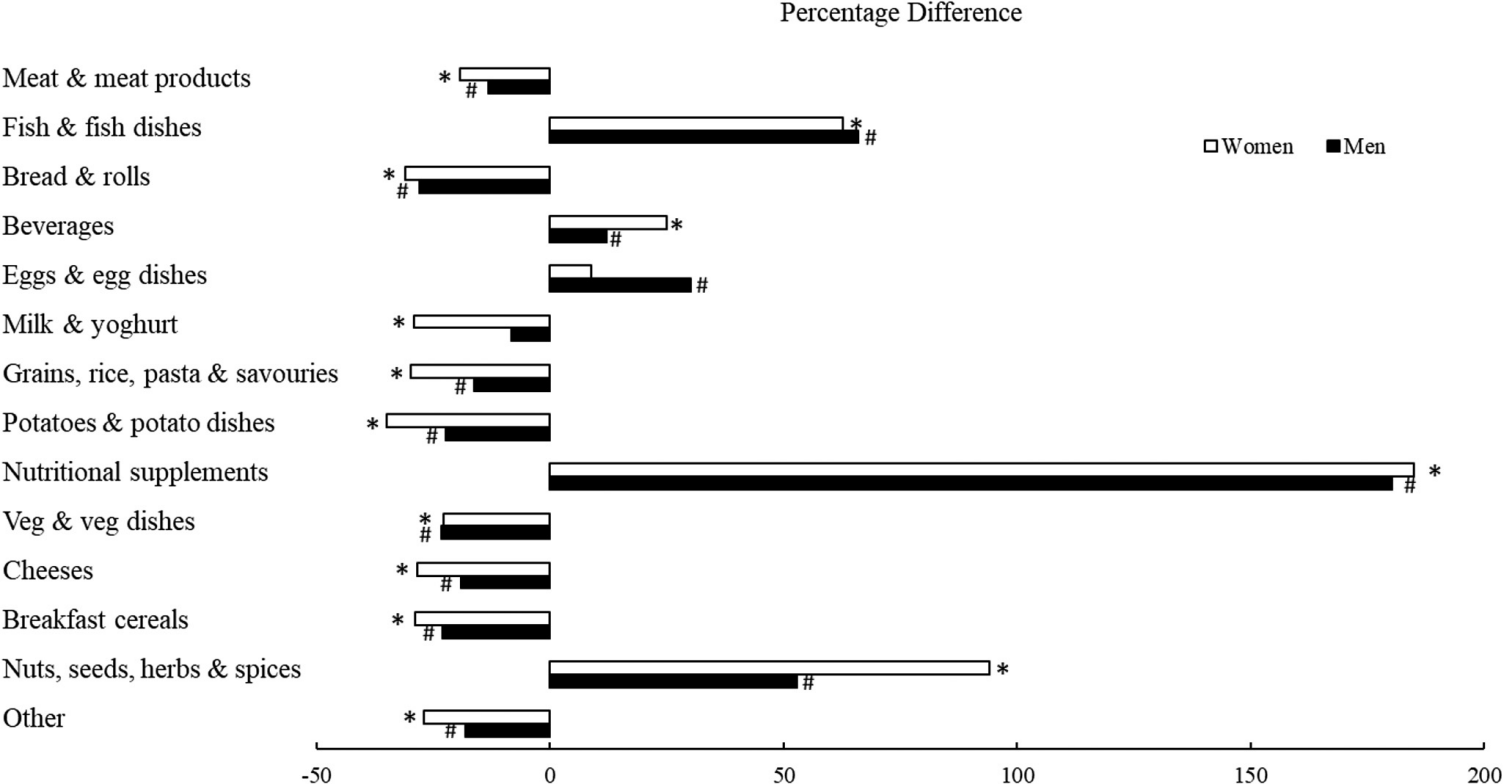
Percentage difference of contributing food groups between low and high consumers of Se for men and women. Low and high Se consumers were compared in terms of percentage contribution from each food group to Se intake with an independent *t* test, separately for men and women. Significant differences (*P* < 0⋅05) in the percentage contribution between low and high consumers are denoted by * for women and # for men. The ‘other’ category included ‘Biscuits, cakes and pastries’, ‘Soups and sauces’, ‘Fruit and fruit dishes’, Sugars, confectionary and preserves’, ‘Ice-creams and desserts’ and ‘Spreading fats and oils’.

Table 4 presents the macro and micronutrient intakes of men and women split by tertiles of low, medium and high Se intake. Mean daily Se intakes ranged from 54 to 111 μg/d between male tertiles, while for women they ranged from 40 to 93 μg/d (*P* < 0·001). Differences in nutrient intake which were common to both genders were intakes of the B vitamins biotin and niacin, which were higher in the high Se consumer groups for both men and women (*P* < 0·05). Intakes of zinc were significantly lower at 9·24 μg/1 0MJ (men) and 7 μg/10 MJ (women) for both genders within the low Se tertiles compared with higher Se consumers. In men, only the low Se tertile group had significantly lower intakes of vitamin B12, vitamin E and iodine compared with higher Se consumers (*P* < 0·05). While a similar stepwise increase in iodine intake was also observed across female tertiles, this result lost significance when adjusted for multiple comparisons. High female Se consumers displayed significantly higher vitamin B6 intakes. Lastly, there was a notable difference in overall dietary quality across the tertiles, with a significant step wise increase in AHEI scores from low to high Se consumers observed in both men and women (ranged from 21 to 26 and 23 to 30, respectively, *P* < 0·001). Fig. 2 displays the main food groups driving the differences between low and high consumers of Se, separately for men and women. The biggest percentage difference in food group contributions between low and high consumers for women, were the ‘nutritional supplements’, ‘nuts, seeds, herbs and spices’ and ‘fish and fish dishes’, with the high Se consumers consuming significantly more of each group at 185, 94 and 63 %, respectively (*P* < 0·001). Although the three groups displaying the largest percentage difference were the same for men at 180, 53 and 66 %, respectively, this difference was only significant for the ‘nutritional supplements’ and ‘fish and fish dishes’ (*P* < 0·001). When men and women within the high Se consumer group were compared statistically, an interesting pattern emerged whereby men displayed higher contribution of ‘grains, rice and pasta’, ‘milk and yogurt’, ‘potatoes and potato dishes’ and ‘meat and meat products’ (*P* < 0·01). In comparison, women in the high Se tertile group had higher contributions from ‘veg and veg dishes’ and ‘fruit and fruit dishes’, ‘beverages’, ‘nutritional supplements’ and ‘nuts, seeds, herbs and spices’ when compared to men in the high Se tertile group (*P* < 0·01).

**Table 4.** Micronutrient intake of men and women split by tertiles of low, medium and high Se consumptions

Nutrient intake	Men				Women			
	Low Se (*n* 246)	Med Se (*n* 247)	High 3 (*n* 247)	*P*-value	Low Se (*n* 253)	Med Se (*n* 254)	High Se (*n* 253)	*P*-value
	Mean ± sd	Mean ± sd	Mean ± sd		Mean ± sd	Mean ± sd	Mean ± sd	
Selenium (µg/d)	53·81^a^ ± 9·16	75·82^b^ ± 5·40	110·86^c^ ± 27·89	**<0·001**	39·61^a^ ± 7·29	58·03^b^ ± 4·95	92·51^c^ ± 31·39	**<0·001**
Energy (kcal)	1838·05^a^ ± 477·53	2412·40^b^ ± 518·29	2761·13^c^ ± 624·62	**<0·001**	1379·92^a^ ± 358·01	1749·76^b^ ± 383·06	1965·38^c^ ± 465·97	**<0·001**
Macronutrients								
Protein (%TE)	16·91^a^ ± 3·67	17·09^b^ ± 3·99	17·56^c^ ± 3·80	**<0·001**	16·71^a^ ± 4·18	16·80^b^ ± 3·37	17·27^c^ ± 3·43	**<0·001**
Carbohydrate (%TE)	45·90 ± 7·70	45·17 ± 7·64	42·80 ± 7·62	0·074	48·58^a^ ± 7·05	47·09^b^ ± 6·32	44·70^c^ ± 6·46	**<0·001**
Total sugar (%TE)	16·77 ± 6·55	17·23 ± 6·53	16·70 ± 5·61	1·000	19·40 ± 6·29	19·06 ± 5·90	18·45 ± 5·60	0·739
Total fat (%TE)	33·04 ± 6·90	33·93 ± 6·18	32·95 ± 6·50	1·000	34·25 ± 7·11	34·23 ± 5·80	34·61 ± 6·06	1·000
Saturated fat (%TE)	13·24 ± 3·65	13·62 ± 3·55	12·63 ± 3·28	0·775	13·66 ± 3·97	13·22 ± 3·31	13·12 ± 3·47	1·000
Monounsaturated fat (%TE)	12·04 ± 2·98	12·30 ± 2·51	12·29 ± 2·84	1·000	12·26 ± 2·74	12·37 ± 2·49	12·60 ± 2·55	1·000
Polyunsaturated fat (%TE)	5·57 ± 2·14	5·75 ± 2·17	5·73 ± 1·85	1·000	6·18 ± 2·90	6·37 ± 2·13	6·62 ± 2·21	1·000
Fibre (g/10 MJ)	21·97 ± 7·57	21·69 ± 6·84	21·22 ± 7·85	1·000	24·90 ± 8·14	25·24 ± 8·15	24·70 ± 8·45	1·000
Vitamins								
Biotin (mg/10MJ)	46·75^a^ ± 31·16	48·81^b^ ± 32·46	60·19^b^ ± 42·46	**<0·001**	53·02^a^ ± 49·69	55·14^a^ ± 41·24	98·41^b^ ± 223·22	**0·013**
Folate (μg/10 MJ)	403·59 ± 184·22	435·71 ± 412·83	422·97 ± 186·15	1·000	516·11 ± 1405·41	442·15 ± 464·40	559·07 ± 708·27	1·000
Niacin (mg/10 MJ)	30·56^a^ ± 13·40	31·00^b^ ± 14·02	36·40^c^ ± 15·41	**<0·001**	31·75^a^ ± 26·70	33·06^a^ ± 44·51	40·06^b^ ± 24·34	**0·003**
Pantothenate (mg/10 MJ)	7·97 ± 6·90	8·31 ± 7·06	9·36 ± 13·25	1·000	8·34 ± 7·45	8·67 ± 6·57	14·49 ± 28·26	0·145
Riboflavine μg/10 MJ)	2·92 ± 5·92	3·06 ± 5·43	3·27 ± 5·26	1·000	3·22 ± 8·48	3·23 ± 5·37	6·56 ± 17·66	0·338
Thiamine (mg/10 MJ)	2·70 ± 5·86	2·76 ± 5·42	3·02 ± 5·04	1·000	2·84 ± 6·53	3·31 ± 7·11	6·83 ± 18·47	0·147
Vitamin B6 (mg/10 MJ)	3·88 ± 5·85	3·92 ± 5·21	4·33 ± 4·74	1·000	3·87^a^ ± 6·37	4·17^a^ ± 5·74	8·66^b^ ± 18·84	**0·007**
Vitamin B12 (mg/10 MJ)	5·64^a^ ± 6·17	6·35^a^ ± 7·72	7·63^b^ ± 6·93	**0·003**	6·26 ± 10·31	6·174 ± 5·44	17·14 ± 82·98	1·000
Vitamin D (μg/10 MJ)	4·72 ± 7·41	5·00 ± 9·62	5·55 ± 5·16	1·000	5·96 ± 7·51	5·56 ± 6·69	7·88 ± 8·55	1·000
Vitamin E (mg/10 MJ)	10·78^a^ ± 6·68	11·83^a^ ± 7·37	15 26^b^ ± 31·26	**0·022**	18·78 ± 49·43	16·28 ± 35·22	22·88 ± 30·11	1·000
Minerals								
Calcium (mg/10 MJ)	1118·93 ± 424·79	1061·99 ± 317·75	1032·69 ± 308·36	1·000	1274·65 ± 563·99	1170·40 ± 480·13	1185·90 ± 472·27	0·789
Iodine (μg/10 MJ)	157·81^a^ ± 68·85	173·40^b^ ± 78·42	174·32^b^ ± 86·25	**0·009**	179·60 ± 84·58	182·30 ± 82·19	201·70 ± 107·40	0·347
Iron (mg/10 MJ)	16·35 ± 17·03	16·16 ± 17·77	15·86 ± 9·56	1·000	18·81 ± 25·75	18·08 ± 23·67	21·50 ± 30·69	1·000
Potassium (mg/10 MJ)	3570·29 ± 760·57	3553·29 ± 635·77	3521·95 ± 661·60	0·989	3921·26 ± 1274·74	3829·45 ± 815·95	3781·52 ± 760·40	1·000
Sodium (mg/10 MJ)	3025·51 ± 734·47	3032·19 ± 690·62	2899·76 ± 638·01	1·000	3054·60 ± 789·52	2936·88 ± 629·86	3026·25 ± 686·73	0·546
Zinc (mg/10 MJ)	9·24^a^ ± 4·69	11·45^b^ ± 3·50	14·11^c^ ± 6·56	**<0·001**	7·00^a^ ± 4·54	8·65^a^ ± 4·70	11·45^b^ ± 8·33	**<0·001**
Diet Quality Score								
AHEI	20·98^a^ ± 8·55	23·71^b^ ± 8·74	26·15^c^ ± 9·85	**<0·001**	22·73^a^ ± 10·82	26·38^b^ ± 8·80	29·54^c^ ± 9·79	**<0·001**

Se, Selenium; AHEI, Alternative Healthy Eating Index.

Differences across tertiles were assessed using GLM adjusting for age and underreporting. *P*-values were Bonferroni corrected by multiplying by the number of traits in the table. *P*-values that exceed 1·0 after correction for multiple testing have been marked down to 1·000. ^a,b^Mean values with unlike superscript letters are significantly different (*P* < 0.05 have been highlighted in bold font).

## Discussion

The present study has shown that the MDI of Se for the total Irish population are above the recommended AI level. Intakes in men are significantly higher than women however, when adjusted for energy, Se intakes were higher in women. The largest contributors to Se intakes were meat and meat products, followed by fish and fish dishes. Despite the MDI of Se for the total population being adequate, 47 % of Irish adults aged 18–90 years were not meeting the AI for Se, and when spilt by gender a higher proportion of women (65 %) compared with men (29 %) did not meet this recommendation. Furthermore, it is clear that the younger men (18–35 years) are driving the higher intakes observed in men when compared with women. In general, higher Se consumers within this cohort have a better overall dietary quality. Regarding overexposure to Se (selenosis), intakes by the highest consumers i.e. those in the 97·5th percentile, were well below the TUL hence, the Irish adult population were not deemed at risk of overexposure to Se.

### Selenium intake

A previous assessment of Se intakes by Irish adults in 2002 by Murphy *et al.* revealed a similar pattern of significantly higher intakes by men in μg/d, while MDI were higher in women when adjusted for energy^(10)^. However, mean daily Se intakes were reported as 52 μg/d, thus there appears to have been a substantial increase in Se consumption by approximately 20 μg/d by the Irish adult population. It is important to note that this earlier cohort excluded over 65 s and is thus not representative of the total population. Interestingly, unlike in the present study which found that men aged 18–35 years (*P* < 0·001) and women aged 50–64 years had the highest intakes of Se, previously it was 36–50-year-olds (both men and women) who displayed significantly higher Se intakes^(10)^. Results from the UK National Diet and Nutrition Survey (NDNS) showed that for the majority of age groups, mean Se intakes were below the reference nutrient intake (RNI) and that 37 % of adults aged 19–64 years and 41 % of >65-year-olds had Se intakes (from all sources) below the LRNI of 40 μg/d^(37)^. According to this report from the Scientific Advisory Committee on Nutrition (SACN), mean Se intakes by the total adult population in the UK are substantially lower than that of the Irish adults included in the present study at 51 μg/d (19–64 years) and 50 μg/d (65+ years)^(37)^. A systematic review of studies exploring selenium intake and status which included results from nineteen European countries, highlighted poor selenium intakes throughout Europe, with a gradient of lower intakes in Eastern countries when compared with their western counterparts, with Poland seeing the lowest consumption^(9)^. Se intakes observed in the current Irish study lie somewhere between that of French and Spanish populations, the highest of which exceeded the AI at 94·4 and 107·1 μg/d for Spanish men and women, respectively. Intakes were perhaps more comparable to that of the French, the highest of which were 64 μg/d. In general, current Irish intakes seem to be more comparable to Western European countries which are reporting similar patterns of Se intake.

### Sources of selenium

In terms of sources of Se in the diet, there has been a shift in the main contributing food groups to Se intake in the Irish population compared with the 2002 study by Murphy *et al.* While ‘meat and meat products’ are still the top contributing food group, ‘bread and rolls’ made a much less contribution, with a 14 % reduction in contribution observed. A possible explanation for this could be the Se content of wheat grains, the Se content of which generally depend on the Se content of the soil in which they are grown^(13,14)^. In the previous study reviewing Irish population intakes of Se, Murphy *et al.* supplemented the nutritional composition data for Irish breads and wheat with data from chemical analysis of Se in Irish and UK breads and wheat^(15,38)^. They reported Irish brown breads as having higher Se levels (8·6–12·9 μg/100 g) than those of white bread (6·6 μg/100 g). They also concluded that Irish flours and breads did not contain as much Se as North American or Canadian flours/breads and contained only slightly more Se compared with those currently used in the UK^(15)^. This may explain the lower contribution of breads and rolls to Se intake seen in the present study since the Se concentration data used here came from the more recently completed TDS which reported lower average values for Se content of 7 μg/100 g for brown bread and rolls and 4 μg/100 g for white bread^(27)^. The variation in the Se content of foods, particularly in wheat and cereal grains, is dependent on the soil and the soils geochemistry and the relatively poor Se content of European soils has been eluded to elsewhere^(13,14)^. This shift in the contribution of food groups to Se intakes since 2002 highlights the need for taking nutrient composition into consideration when sourcing raw commodities. Although more locally sourced commodities are favourable in terms of sustainability, including them in our diets may impact on nutrient intakes. Overall, the main contributing food groups to Se intake reported here are i.e. meat, fish, dairy, bread and cereals are in keeping with many other European populations^(9,39)^. A similar pattern of contributing food groups was observed within the UK, with ‘beverages’ stated as one of the top contributing food groups, which is in concurrence with results from the present study. However, it should be noted that beverages overall are relatively low in Se content when reviewed per 100 g^(37)^. Therefore, the high contribution from beverages observed in the present study and others, which includes teas, water (flavoured/unflavoured), alcohol, etc., is perhaps more reflective of the volume of consumption of this food group rather than a high Se content of these types of beverages.

### Dietary quality

The comparison of micronutrient intakes between low medium and high Se tertile members, revealed differences in intakes of micronutrients other than Se. Men in the low Se tertile also had low intakes of vitamin B12, vitamin E and iodine compared with the higher tertiles. Intakes of zinc were significantly lower for both genders within the low Se tertiles compared with the higher Se tertiles. The recommended daily intake of vitamin D by adults is 10 μg/d^(40)^, while for older free living (≥65 years; not housebound) adults in Ireland, is 15 μg/d^(41)^. Although no significant difference between vitamin D intakes was apparent across tertiles in the present study, in general vitamin D intakes appear to be suboptimal at each tertile level, for both genders which is in keeping with results from previous studies which have examined the Irish adult population intakes of vitamin D^(42,43)^. It has been suggested that meat, which is the major contributing food group to Se intake in the present study, also makes a significant contribution to intakes of vitamin B12, vitamin D and zinc in other populations^(44)^. It, therefore, seems logical that low Se intakes were coupled with low intakes of these other important micronutrients. Using the AHEI index, a score was calculated for each participant based on their fruit, vegetable, nut and soya protein, and cereal fibre consumption, all of which achieved a max score of 10 if recommendations were met. The score also factored in the ratio of white to red meat, the ratio of polyunsaturated to saturated fat, trans fat intake, alcohol consumption as well as the duration of multivitamin use. Participants who had an overall higher AHEI score also had more optimal levels of Se consumption. In general, the poorer dietary quality observed in the low Se consumer tertile appears to be having a detrimental effect on the intakes of various micronutrients. The finding that women within the high Se tertile group had higher contributions from fruit, vegetables, nuts and seeds but lower contributions from animal products like, milk, yoghurt and meat when compared with their male counterparts, suggests that a diet rich in selenium is achievable with a plant-based style diet. This is particularly pertinent in the current climate as we as a nation explore the benefits of more sustainable, plant-based style diets.

### Selenium status

Although not measured in the present study, monitoring micronutrient status is potentially even more important than estimating population intakes, as it evaluates the amount of biologically active nutrient in the body; however, it is not always feasible to do so. Suboptimal Se status may reduce the activity of seleno-proteins and subsequently affect the efficiency of the physiological processes that these seleno-proteins are involved in, this may lead to adverse health effects such as reduced oxidative capacity, altered fertility and greater susceptibility to CVD. Adequate Se status is essential for optimal immune function, and Se status has been shown to be inversely proportional to mortality risk from diseases like sepsis^(45)^. A recent study involving thirty-three patients suffering from COVID-19 showed them to be deficient in total serum Se, as well as having low concentrations of the Se transporter. This study also found that Se status was higher in surviving *v.* non-surviving COVID patients^(46)^. The poorer consumption of vitamin D and zinc observed in the low Se consumer group is a worrying trend given the important roles of these micronutrients in the maintenance of immune function. There is some evidence to suggest that early intervention to achieve adequate levels of vitamin D, Se and zinc as an adjuvant therapy, may alleviate the escalation of COVID-19^(47)^. Due to the observational nature of these studies and relatively small sample sizes, it is difficult to deduce the mechanisms involved however, one hypothesis is that the antioxidant function of seleno-proteins prevents tissue damage often seen following viral infections caused by inflammation. Khatiwada and Subedi have proposed that the mechanism for the protective effect of Se against diseases like coronavirus is via the oxidative stress-reducing capabilities that Se exhibits in those with adequate status^(48)^. Therefore, it is crucial that we continue to monitor the intakes of this important micronutrient by those at risk of, or more susceptible to adverse effects of low-grade inflammation e.g. the elderly and those with metabolic diseases. A study which measured the selenium status of a cohort of healthy Irish adults has suggested that serum selenium levels were on average 76 ± 21 μg/l, and therefore not at the level (95 μg/l) deemed sufficient to maintain glutathione peroxidase activity^(49,50)^. Although the findings of this study are very interesting, it was conducted in a relatively small cohort of 91 individuals recruited from one Irish city^(49)^. Future studies measuring the levels of serum selenium or one of the many seleno-proteins e.g. GPX3 or SEPP1 in a larger, nationally representative cohort would be beneficial in order to compliment estimates of population dietary intake.

### Strengths and limitations

One of the main strengths of the present study is the comprehensive dietary data derived from the 4-d weighed food diary collected for this nationally representative sample. Data were obtained from current food composition tables and databases, brand information and published papers, to ensure the most up-to-date Se concentrations were applied in a bid to achieve the most accurate estimates of Se intake. However, due to the self-reporting nature of dietary assessments, there is difficulty in assuring 100 % accuracy of estimates of Se intake, this is due in part to the presence of under-reporters who, although were not excluded from the entire analysis, were adjusted for during statistical analysis where appropriate. Furthermore, use of the AI levels to assess adequacy of population intakes of Se comes with some inherent limitations due to varying methods used to set an AI. An AR for Se, which is usually the more appropriate indicator of measuring inadequacies at the population level, has not yet been set by EFSA due to lack of sufficient evidence from human studies^(31)^. Lastly, the lack of measurement of Se status is a limitation of the present study. Future nutrition surveys involving the Irish adult population would benefit from the measurement of a biomarker of Se status.

## Conclusion

The average intake of Se in the Irish adult population is above the AI level recommended by EFSA. However, since meat and fish are major contributors to Se intakes in Irish adults, it will be important to continue monitoring intakes, especially with plant-based diets growing in popularity and simultaneous reductions in meat consumption. Thus, as the food environment continues to change and shift to include more sustainably sourced raw commodities, the present study provides a rational for the continued monitoring of Se intakes by Irish adults and for the development of public health campaigns to promote the consumption of Se-rich foods by the population, in general, and, in particular, those at risk.

## Supplementary material

For supplementary material accompanying this paper visit https://doi.org/10.1017/jns.2023.23. click here to view supplementary material
