# Selenium intakes in the Irish adult population – CORRIGENDUM

**DOI:** 10.1017/jns.2023.35

**Published:** 2023-04-12

**Authors:** Maria Buffini, Anne P. Nugent, Janette Walton, Albert Flynn, Breige A. McNulty

**Affiliations:** 1 UCD Institute of Food and Health, School of Agriculture and Food Science, University College Dublin, Belfield, Dublin 4, Republic of Ireland; 2 Institute for Global Food Security, Queens University Belfast, Belfast, Northern Ireland, UK; 3 School of Food and Nutritional Sciences, University College Cork, Cork, Republic of Ireland; 4 Department of Biological Sciences, Munster Technological University, Cork, Republic of Ireland

In Table 2 the arrow before the letters ‘TUL’ is pointing downwards. The arrow should be pointing upwards.

The correct table is shown below.

**Table 2.** Percentage of population groups with mean daily Se intakes (μg/day from all sources) below the adequate intake and lower reference nutrient intake and above the tolerable upper limit.

	Total Population*	Men	Women	Men				Women			
	(n = 1051)	(n = 523)	(n = 528)	18–35y	36–50y	51–64y	≥65y	18–35y	36–50y	51–64y	≥65y
				(n = 207)	(n = 143)	(n = 98)	(n = 75)	(n = 170)	(n = 165)	(n = 106)	(n = 87)
% ↓AI	46·81	28·68	64·77	20·77	34·27	30·61	37·33	57·06	67·27	66·98	72·41
% ↓LRNI	4·09	0·96	7·2	0	2·1	0	2·67	7·65	5·45	6·6	10·34
% ↑TUL	0	0	0	0	0	0	0	0	0	0	0

AI (adequate intake), LRNI (lower reference nutrient intake) and TUL (tolerable upper limit) are 70 μg/day, 40 μg/day and 300 μg/day respectively^(31,32,33)^. *Under reporters excluded.

