# Supplementary material for: Selenium intakes in the Irish adult population
Source: J Nutr Sci. 2023 Mar 13;12:e35. doi: 10.1017/jns.2023.23 (PMC10052560; doi:10.1017/jns.2023.23)
Supplement: Supplementary file 1 [file S204867902300023Xsup001.docx]

**Supplementary Table 1.** Mean daily intake (µg/10MJ total energy) from all sources (food and supplements) and from food sources only split by gender and by age category

Significant differences (p<0.01) between age groupings within the same gender compared by ANOVA are denoted by superscript letters, while the * denotes a significant difference between men and women compared by t-test.
